# Inhibition of KCTD10 Affects Diabetic Retinopathy Progression by Reducing VEGF and Affecting Angiogenesis

**DOI:** 10.1155/2022/4112307

**Published:** 2022-10-26

**Authors:** Yun Feng, Cong Wang, Guangwei Wang

**Affiliations:** ^1^Department of Ophthalmology, Changsha Central Hospital, University of South China, Changsha 410004, China; ^2^Department of Geriatrics, The Third Hospital of Changsha, Changsha 410015, China; ^3^Key Laboratory of Brain and Neuroendocrine Diseases, College of Hunan Province, Huaihua 418000, China; ^4^Biomedical Research Center, Hunan University of Medicine, Huaihua 418000, China

## Abstract

**Aim:**

We purposed to evaluate the KCTD10 effects of angiogenesis in diabetic retinopathy (DR).

**Methods:**

We induced a DR cell model using high glucose (HG) treatment of HRECs and ARPE-19 cells. A DR rat was established by injecting streptozotocin. Small interference RNA targeted KCTD10 (si-KCTD10) was used to mediate KCTD10 inhibition in cell and animal models. The roles of KCTD10 on cell viability, apoptosis, angiogenesis, and related proteins (VEGF and HIF-1*α*) were observed by RT-qPCR, Western blot, CCK-8 assay, TUNEL staining, tube formation assay, ELISA, and immunohistochemistry assay.

**Results:**

KCTD10 expression was upregulated in DR cells and retinal tissue of DR rats. Treatment of the cells with si-KCTD10 increased cell viability and decreased apoptosis and angiogenesis in DR cells. Inhibition of KCTD10 could reduce the expression of VEGF and HIF-1*α* in DR cells. Furthermore, KCTD10 inhibition reduced VEGF levels in the retinal tissue of DR rats.

**Conclusion:**

This work showed that inhibition of KCTD10 relieved angiogenesis in DR.

## 1. Introduction

Diabetic retinopathy (DR) is a microvascular disease in the retina of diabetes mellitus (DM) patients. DR can cause visual impairment and even blindness in patients, which has become one of the main causes of blindness [1, 2]. Numerous studies have recognized that DR results from chronic hyperglycemia, which is characterized by leaky retinal vasculature, retinal ischemia, retinal inflammation, and neovascularization [3]. VEGF plays a dominant role in the initiation and development of DR, and its transcriptional expression in the retina is controlled by hypoxia-inducible factor-1*α* (HIF-1*α*) [4, 5]. VEGF increases vascular permeability by inducing the phosphorylation of claudin proteins such as occludin [6]. Furthermore, VEGF promotes endothelial cell proliferation as an angiogenic factor by activating mitogen-activated protein (MAP), leading to retinal angiogenesis [7–9]. VEGF blockers have been developed as drugs for the treatment of retinal neovascularization [10]. However, VEGF intervention has some limitations, such as producing other angiogenic factors and proinflammatory mediators that may render anti-VEGF therapy ineffective and cause resistance [10]. There is an urgent need to investigate the mechanisms leading to DR.

The human family of potassium (K^+^) channel tetramerization domain (KCTD) proteins has a total of 25 members that have been implicated in neurological diseases, obesity, and the regulation of specific oncogenic pathways and can be used as therapeutic targets or diagnostic/prognostic markers for diseases [11]. Dysregulations in KCTD genes have been related to cancer initiation [12]. Early studies found that KCTD10 (KCTD-containing 10) may be involved in DNA synthesis and cell proliferation [13]. KCTD10 interacted with the small subunits of DNA polymerase delta and PCNA, affecting DNA replication, repair, and cell cycle progression [14, 15]. Meanwhile, KCTD10 can act as a substrate-recognition receptor for Cullin-3 to regulate protein ubiquitination and play different roles in endothelial barrier formation, primary cilia formation, plasma membrane dynamics, cell proliferation, and immune responses [16]. Interestingly, VEGF induced the expression of KCTD10, which played a key role in the angiogenesis and heart development of mammalian embryos by inhibiting notch signals [17]. Upregulation of KCTD10 inhibited notch signaling to prevent congenital heart disease and cardiac dysplasia [18]. An additional K^+^ channel, the purinergic P2X7 receptor, plays a role in the pathogenesis of DR [19, 20].

At present, the functions of KCTD10 in DR angiogenesis remain unknown. This study attempted to explore the mechanism of action of KCTD10 in angiogenesis in DR and to provide a rationale for the development of more effective antiangiogenic therapies for DR.

## 2. Methods

### 2.1. Cell Culture

Human retinal microvascular endothelial cells (HRECs) were obtained from Procell (Wuhan, China). HRECs were cultured in a proprietary medium containing 1% penicillin, 1% streptomycin, and 5% fetal bovine serum (FBS). An ARPE-19 cell line was provided by Abiowell (Changsha, China). ARPE-19 cells were maintained in DMEM/F12 medium (Hyclone, Beijing, China) containing 1% penicillin, 1% streptomycin, and 10% FBS. HRECs and ARPE-19 cells were seeded at 1 × 10^4^ in 6-well plates and treated with normal glucose (5.5 mmol/L) as a control or with high glucose (HG, 25 mmol/L). Normoxic conditions for 72 h to simulate the early stages of DR [21]. The medium was changed daily to eliminate metabolic byproducts and provide the cells with nutrients. An HG-induced DR cell model was successfully established.

### 2.2. Cell Transfection

Small interfering RNA specifically targeted KCTD10 (si-KCTD10, 5′-CCCTACAACAGAACAAAGA-3′), and the negative control (si-NC, 5′-UUCUCCGAACGUGUCACGU-3′) was designed from HonorGene (Changsha, China). According to the manufacturer's protocols, ARPE-19 cells were transfected with si-KCTD10 and si-NC using Lipofectamine 3000 (Invitrogen). After 48 h of treatment, the cells were harvested for subsequent experiments [22].

### 2.3. Quantitative Reverse Transcription PCR (RT-qPCR)

According to previous research, mRNA levels of VEGF and KCTD10 in cells and retinal tissues were performed by RT-PCR [23]. Total RNAs were extracted from cells and retinal tissues using the TRIzol method (Thermo, USA). The RNA was synthesized into cDNAs using a cDNA reverse transcription kit (CW2569, CWBIO, China). We performed RT-qPCR to examine mRNA levels using a fluorescence quantitative PCR instrument (QuantStudio1, Thermo). Using GAPDH as the inside reference gene, the relative mRNA levels were computed by the 2^−ΔΔCt^ method. The primer sequences were shown in Supplemental Table 1.

### 2.4. Western Blot

Total protein from cells and retinal tissues was extracted using the RIPA kit (AWB0136, Abiowell) [24]. Proteins were segregated using 10% SDS polyacrylamide gel electrophoresis. Isolated protein was loaded onto the nitrocellulose filter membrane by electrotransfer, then blocked by PBST containing 5% Bovine albumin with 2.5 h. Primary antibodies KCTD10 (27279-1-AP, 1: 1000, Proteintech), VEGF (19003-1-AP, 1: 500, Proteintech), HIF-1*α* (20960-1-AP, 1: 5000, Proteintech), *β*-actin (66009-1-Ig, 1: 6000, Proteintech) were reacted with the membranes for 2 h. The secondary antibodies reacted with the membranes for 2 h. Then western blot band was determined by SuperECL Plus (AWB0005, Abiowell) and a chemiluminescence imaging system (Chemiscope 6100, Clinx, China).

### 2.5. Cell Viability Detection

According to previous research, cell viability was determined using the Cell Counting Kit-8 (CCK-8) assay [25]. Cells were digested, counted, and seeded in a 96-well plate (5 × 10^3^ cells/well, 100 *μ*L). After the cells culturing adherent, the treatment was carried out according to the above method for a corresponding time, and then 10 *μ*L of CCK-8 solution (NU679, DOJINDO) was added to each well. After incubation at 37°C and 5% CO_2_ for 4 h, the absorbance (OD) value at 450 nm was measured with a Bio-Tek microplate reader (MB-530, HEALES).

### 2.6. Terminal Deoxynucleotidyl Transferase-Mediated dUTP-Biotin Nick-End Labeling (TUNEL) Staining

The apoptosis of ARPE-19 cells was evaluated by the TUNEL apoptosis detection kit (40306ES50, YEASEN, China) [25]. Briefly, after the cell slides were fixed with 4% paraformaldehyde for 0.5 h, 100 *μ*L proteinase K solution was added dropwise, and the reaction was performed at 37°C for 20 min. Then, the slides were added 100 *μ*L of 1 × equilibration buffer and incubated at 25°C for 10 min. Finally, the slide was added 50 *μ*L TdT buffer and incubated at 37°C for 1 h without light. The cell nucleus was stained with DAPI at 37°C for 10 min. Images were viewed and collected by a fluorescence microscope (BA410T, Motic).

### 2.7. Tube Formation Assay

The angiogenic capacity of HRECs was observed by tube formation assay [26, 27]. Briefly, HRECs were treated with an ARPE-19 cell culture medium for 5 h. The different groups were determined by the media used: control medium, Model medium, KCTD10-siRNA transfected control (NC-siRNA) medium, and KCTD10-siRNA transfected medium from ARPE-19 cells. HRECs (1 × 10^4^ cells per well) were harvested by digestion and plated onto 96-well glass slides precoated with Matrigel (356234, Biocoat) and incubated at 37°C for 4 h. The images were photographed under a microscope (DSZ2000X, CNMICRO, China).

### 2.8. Enzyme-Linked Immunosorbent Assay (ELISA)

VEGF expression was detected by an ELISA kit (KE00085, Proteintech) [28]. 1 × 10^6^ cells were collected, a certain amount of 1 × PBS was added, the cell membrane was disrupted by ultrasonication, and then centrifuged at 10000 rpm at 2–8°C for 10 min. The supernatant was collected and assayed for VEGF content. The OD value was detected by a multifunctional enzyme label analyzer (MB-530, HEALES). VEGF concentration was calculated by forming the standard curve through the provided constant value.

### 2.9. Animal Experiment

SPF-grade male Sprague–Dawley (SD) rats (weighing about 250 g) were ordered from Hunan SJA Laboratory Animal Co., Ltd. After one week of adaptive feeding; the SD rats were stochastically divided into the following 4 groups: control group (10 rats), DR group (10 rats), DR + KCTD10 interference control group (si-NC, 10 rats), and DR + KCTD10 interference group (si-KCTD10, 10 rats). The rats in DR, si-NC, and si-KCTD10 groups were treated with streptozotocin (STZ) at 65 mg/kg [21]. Control rats were not subjected to any manipulation. The night before the intervention, the rats were fasted (12–16 h). The rats were randomly tested for blood sugar before fasting. Random blood glucose was measured seven days after injection, and rats with blood glucose higher than 16.7 mmol/L were selected in the formal experiment. After 4 weeks of STZ-induced diabetes, the si-KCTD10 group received an intravitreal injection of 200 nmol/200 *μ*L (one-time injection) of KCTD10-siRNA, and the si-NC group received the same dose of NC-siRNA injection (one-time injection) [29]. One week after injection, one eyeball of 6 rats in each group was taken and soaked in paraformaldehyde for immunohistochemistry assay, and the retinal tissue of the other eyeball was separated for western blot and RT-qPCR experiments. This study was approved by the Medical Ethics Committee of Changsha Central Hospital (No. 201996). The animal experiments adhered to institutional guidelines for the humane treatment of animals, the Principles of Laboratory Animal Care (National Institutes of Health, Bethesda, MD, USA), and the ARVO Statement for the Use of Animals in Ophthalmic and Vision Research.

### 2.10. Immunohistochemistry Assay

The retinal tissues of rats were collected, embedded in paraffin, sliced, and baked at 68°C for 20 min. Following routine xylene deparaffinization and graded alcohol dehydration, sections were left at 25°C for 15 min and added to goat serum blocking solution for 20 min. Then the sections were incubated with antibody KCTD10 (PA5-53138, 1: 100, ThermoFisher) and VEGF (19003-1-AP, 1: 100, Proteintech) at 37°C for 1 h. Next, the sections were incubated with the secondary antibody at 37°C for 1 h. DAB kit (ZSGB-BIO) was used for color development. After hematoxylin staining, sectors were dehydrated, hyalinized, and observed under a microscope (BA410T, Motic).

### 2.11. Statistical Analysis

Each experiment was conducted at least three times. Data were displayed as means ± standard deviations. The data were analyzed as described previously [30]. Statistical analysis between more than two groups and between two groups was performed using one-way analysis of variance (one-way ANOVA) and Student's *t*-test, respectively. It was considered significant when *P*-value <0.05.

## 3. Results

### 3.1. Expression of KCTD10 and VEGF in DR Cells

**Figure 1:**
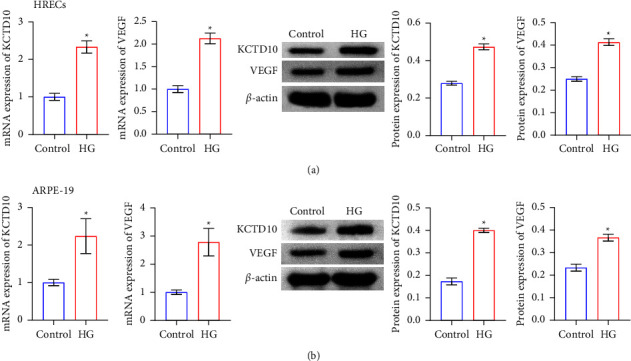
Expression of KCTD10 and VEGF in DR cells. (a) KCTD10 and VEGF expression in HRECs was identified by RT-qPCR and western blot. (b) KCTD10 and VEGF levels in ARPE-19 were determined by RT-qPCR and Western blot. ^∗^*P* < 0.05 versus the control group.

KCTD10 and VEGF expression was examined in HRECs and ARPE-19 cells subjected to HG stimulation. RT-qPCR results showed the expressions of KCTD10 and VEGF were both upregulated in HRECs and ARPE-19 cells subjected to HG stimulation (Figures 1(a) and 1(b)). Meanwhile, western blot results consistently demonstrated that KCTD10 and VEGF levels at HRECs and ARPE-19 cells with HG treatment were upregulated in contrast to the control group (Figures 1(a) and 1(b)).

### 3.2. KCTD10 Inhibition Attenuated HG-Induced ARPE-19 Cell Damage

**Figure 2:**
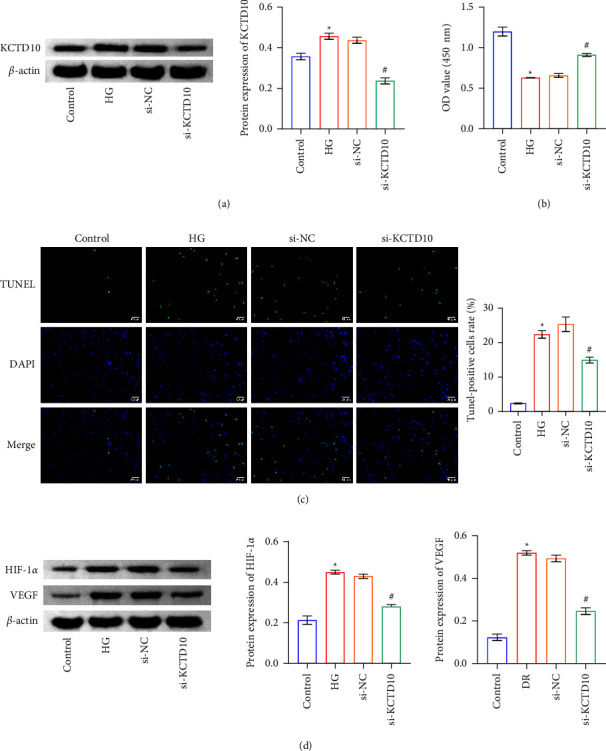
KCTD10 inhibition attenuated HG-induced ARPE-19 cell damage. (a) Western blot detection of KCTD10 protein expression. (b) CCK-8 assay detection of cell proliferation. (c) The cell apoptosis was observed by TUNEL staining. (d) Western blot detection of HIF-1*α* and VEGF expression. ^∗^*P* < 0.05 versus the control group. ^#^*P* < 0.05, versus the si-KCTD10 group.

We investigated the roles of KCTD10 on ARPE-19 cells under high glucose conditions by si-KCTD10 transfection. As shown in Figure 2(a), si-KCTD10 transfection significantly suppressed KCTD10 levels in ARPE-19 cells. Meanwhile, KCTD10 inhibition restored HG stimulation-induced decrease in ARPE-19 cell viability (Figure 2(b)). TUNEL staining showed that KCTD10 inhibition restored the increased ARPE-19 cell apoptosis induced by HG stimulation (Figure 2(c)). Western blot assays revealed that compared to the si-NC group, the levels of HIF-1*α* and VEGF were significantly reduced in the si-KCTD10 group (Figure 2(d)). These data suggested that KCTD10 inhibition restored HG-induced cell viability and hampered apoptosis and the levels of HIF-1*α* and VEGF in ARPE-19 cells.

### 3.3. Inhibition of KCTD10 Regulated HRECs Angiogenesis

**Figure 3:**
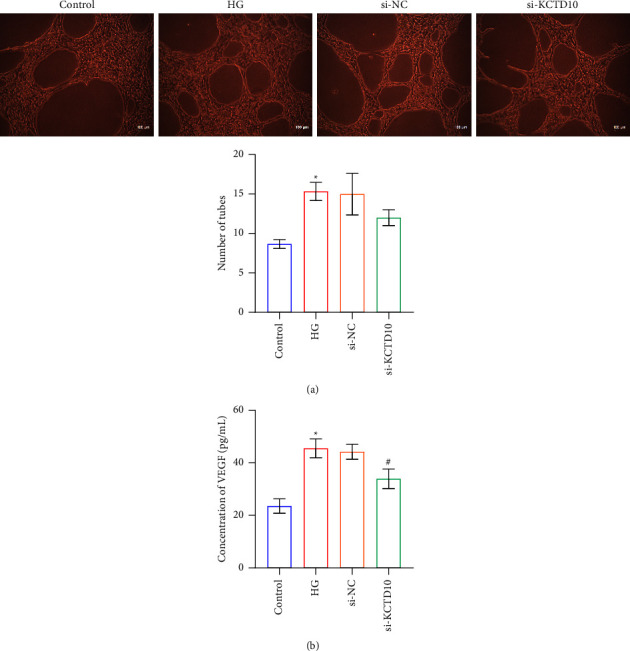
Inhibition of KCTD10 regulated HRECs angiogenesis. (a) The angiogenesis of cells was observed by tube formation assay. (b) VEGF expression of cells was observed by ELISA. ^∗^*P* < 0.05 versus the control group. ^#^*P* < 0.05, versus the si-KCTD10 group.

HRECs were cultured with a cell culture medium obtained from HG-induced ARPE-19 cells and exhibited an increased number of lumen formations relative to controls. These angiogenic changes were partially reversed by HRECs cultured in an ARPE-19 cell medium transfected with si-KCTD10 (Figure 3(a)). VEGF expression was elevated in HRECs cultured in a cell culture medium obtained from ARPE-19 cells induced by HG in contrast to the control group (Figure 3(b)). HRECs cultured with ARPE-19 cell medium of si-KCTD10 transfection partially reversed VEGF expression (Figure 3(b)).

### 3.4. Expression of KCTD10 and VEGF in DR Animal Model

**Figure 4:**
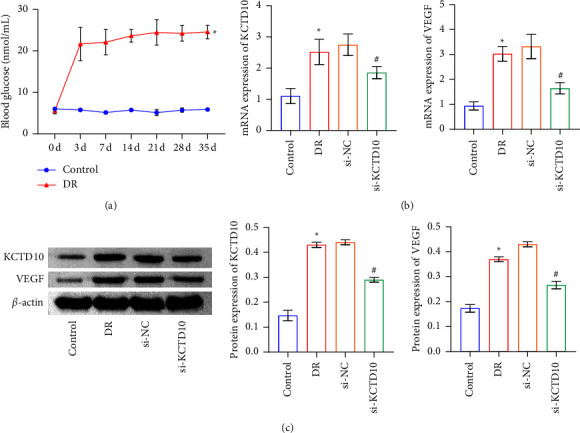
Expressions of KCTD10 and VEGF in DR animal model. (a) Changes in blood glucose levels with a duration of STZ intervention. (b), (c) KCTD10 and VEGF levels in the retinal tissues were identified by RT-qPCR and western blot detection. ^∗^*P* < 0.05 versus the control group. ^#^*P* < 0.05, versus the si-KCTD10 group.

Next, we established a DR animal model. The blood glucose of the DR group was significantly increased in contrast to the NC group, indicating that the DR model was successfully established (Figure 4(a)). The expressions of KCTD10 and VEGF in retinal tissues were explored by RT-qPCR and Western blot. The results demonstrated that compared to the control group, KCTD10 and VEGF were elevated dramatically in the DR group (Figures 4(b) and 4(c)). KCTD10 expression was further silenced, and the results were discovered that KCTD10 had a good inhibition efficiency (Figures 4(b) and 4(c)). Simultaneously, VEGF expression decreased with the decrease of the expression of KCTD10 (Figures 4(b) and 4(c)).

### 3.5. Distribution of KCTD10 and VEGF in Retinal Tissue of DR Animal

**Figure 5:**
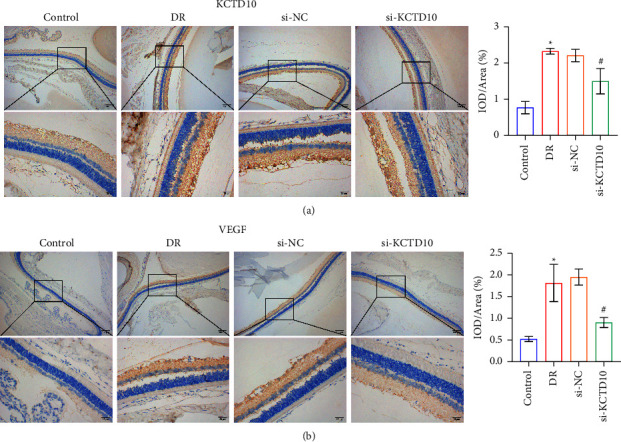
Distribution of KCTD10 and VEGF in retinal tissue of DR animal. (a), (b) The expression of KCTD10 and VEGF in the retinal tissue of DR rats was identified by immunohistochemical assay. ^∗^*P* < 0.05 versus the control group. ^#^*P* < 0.05, versus the si-KCTD10 group.

Next, we detected the distribution and expression of KCTD10 and VEGF in retinal tissue by immunohistochemistry assay. The results indicated that KCTD10 and VEGF were highly expressed in the cell membrane and cytoplasm and elevated in the DR group (Figures 5(a) and 5(b)). After injection of si-KCTD10, the results indicated that KCTD10 was inhibited in the DR rats. Moreover, VEGF expression decreased with the decrease of the level of KCTD10 (Figures 5(a) and 5(b)).

## 4. Discussion

Our work found that KCTD10 and VEGF were augmented in HRECs and ARPE-19 cells under HG stimulation. Silencing KCTD10 in HG-induced HRECs, the cell damage was significantly reduced, and the expressions of VEGF and HIF-1*α* were reduced considerably. Furthermore, silencing of KCTD10 inhibited HG-induced endothelial cell angiogenesis. Moreover, we found that silencing KCTD10 suppressed VEGF expression in the retina of DR rats.

Hyperglycemia is the primary driver of DR, resulting in severe metabolic and biochemical abnormalities [31, 32]. Retinal pigment epithelial (RPE) cells are an essential portion of the outer blood-retinal barrier and may control the flow of biomolecules to and from the retina selectively, so they are hyperglycemic sensitive [33, 34]. Under HG conditions, changes in cytokines in RPE cells trigger a series of intracellular signaling and induce cell damage and neovascularization [35, 36]. Our results showed that HG-induced RPE cell viability was significantly reduced, and apoptosis was boosted. Additionally, HG-induced RPE medium treatment of endothelial cells could increase their angiogenesis. The above results indicated that HG treatment of RPE cells was an important cell model for studying DR angiogenesis.

KCTD10 has been linked to obesity, diabetes, and atherosclerosis [37, 38]. According to our findings, HGstimulation increased KCTD10 expression in HRECs and ARPE-19 cells. In addition, the animal model of DR was established by injecting STZ, and it was found that KCTD10 was increased in the retina of DR rats. Further silencing of KCTD10 could reverse HG-induced RPE cell apoptosis. These data disclosed that KCTD10 might be an important marker of DR and participate in the progression of DR.

Knockdown of KCTD10 resulted in delayed development and impaired maturation of cardiomyocytes [39]. This suggests that inhibiting KCTD10 may have some negative effects on normal cells. We speculate that the regulatory mechanisms of KCTD10 are different under normal and pathological conditions, leading to changes in its functional effects on cells, which require further study.

Previously data showed that VEGF could be produced by RPE cells, endothelial cells, and pericytes of retinal vessels. RPE cells played a more important role in VEGF production [40]. Studies found that targeted inhibition of VEGF and HIF-1*α* expression could inhibit pathological retinal angiogenesis in DR [41]. Moreover, the activation of VEGF was modulated by HIF-1*α* [42]. HIF-1*α* and its target genes have been linked to retinal neovascularization production in many studies, and inhibiting HIF-1*α* might result in more successful DR therapy [43, 44]. Our study found that VEGF and HIF-1*α* were markedly augmented in HG-stimulated ARPE-19 cells, and KCTD10 inhibition reversed this phenomenon. Furthermore, silencing KCTD10 could reduce VEGF expression in the retinal tissue of DR rats. Meanwhile, silencing of KCTD10 inhibited HG-induced angiogenesis in ARPE-19 medium-treated HRECs. These results suggested that silencing KCTD10 might inhibit angiogenesis in DR via inhibiting VEGF and HIF-1*α* expression.

Our study is the first to show that inhibition of KCTD10 increased cell viability and decreased apoptosis and angiogenesis in DR cells. Inhibition of KCTD10 reduced the expression of VEGF and HIF-1*α* in DR cells. This suggests that KCTD10 may be one of the important factors in the progression of DR. KCTD10 can regulate Notch signaling [15]. Notch signaling is associated with altered vascular permeability in DR [38]. However, whether KCTD10 affects DR by regulating Notch signaling remains further investigated. Furthermore, the mechanism of action of KCTD10 on VEGF and HIF-1*α* is rather complex. More experimental data are needed to prove its specific regulatory mechanism. These are the limitations of our work. In the next study, we will verify the downstream mechanism of KCTD10 in DR and the direct target molecules by combining animal and cell models.

## 5. Conclusion

Our study showed that KCTD10 might participate in the development of DR. Inhibition of KCTD10 expression could reduce the expression of VEGF and HIF-1*α* and inhibit angiogenesis in DR.

## Data Availability

The data used to support the findings of this study are included within the article.

## Supplementary Materials

Supplementary Materials Supplemental Table 1: Primer sequences.
