# Supplementary material for: Inhibition of KCTD10 Affects Diabetic Retinopathy Progression by Reducing VEGF and Affecting Angiogenesis
Source: Genet Res (Camb). 2022 Oct 26;2022:4112307. doi: 10.1155/2022/4112307 (PMC9629933; doi:10.1155/2022/4112307)
Supplement: Supplementary Materials — Supplemental Table 1: Primer sequences. [file 4112307.f1.docx]

**Supplemental Table 1. Primer sequences**

| Gene | Sequences (5'-3') |
| --- | --- |
| *H-GAPDH* | forward: ACAGCCTCAAGATCATCAGC |
|  | reverse: GGTCATGAGTCCTTCCACGAT |
| *R-GAPDH* | forward: ACAGCAACAGGGTGGTGGAC |
|  | reverse: TTTGAGGGTGCAGCGAACTT |
| *H-VEGF* | forward: TGCTCTACTTCCCCAAATCACT |
|  | reverse: ACTCACTTTGCCCCTGTCG |
| *R-VEGF* | forward: CCTCGTCCTCTCCCTACCCCACT |
|  | reverse: TCCTGCCCCATTGCTCTGTACCTT |
| *H-KCTD10* | forward: GCGGCCCTACAAAACAAAGA |
|  | reverse: ACAGGACCCTGGTATATGAGT |
| *R-KCTD10* | forward: TTAAGAAGTGTCCCTCTGCGG |
|  | reverse: AACACCCAGCTCACTCAAGG |
